# Supplementary material for: Isolation and Characterisation of Streptococcus spp. with Human Milk Oligosaccharides Utilization Capacity from Human Milk
Source: Foods. 2024 Apr 23;13(9):1291. doi: 10.3390/foods13091291 (PMC11083076; doi:10.3390/foods13091291)
Supplement: Supplementary file 1 [file foods-13-01291-s001.zip › foods-2951646-supplementary.pdf]

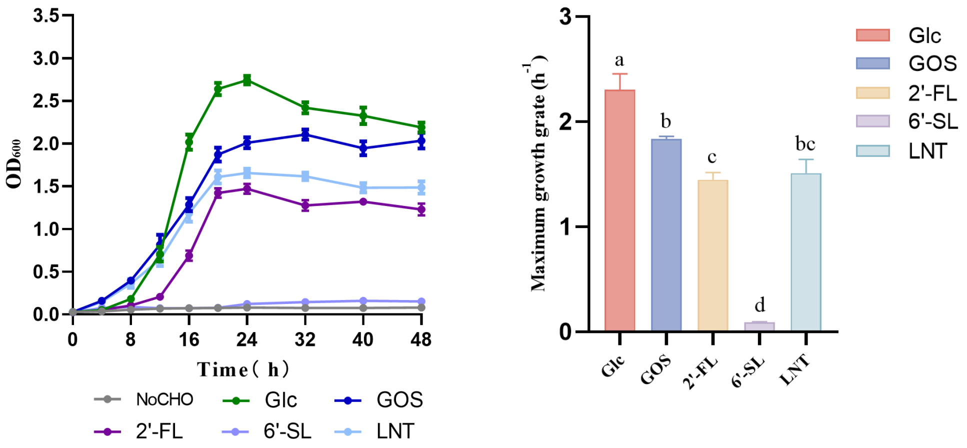

(**a**) (**b**)

Figure S1 The growth curve and maximum growth rate of *B. longum* subsp. *infantis* ATCC 15697; (a) Growth curve; (b) Maximum growth rate. The carbohydrates were denoted by their abbreviation. No Carbohydrate (NoCHO), Glucose (Glc). Each data point reflected the average of three replicates, with the error bars denoting standard deviation. The letters a, b, c, and d indicated statistically significant variances among groups distinguished by different letters (P<0.05).

Table S1: The 16S rRNA sequences of *Streptococcus* sp. strains.

Strains	16S rRNA Sequences
Streptococcus sp. 21WXBC0057M1	>21WXBC0057M1_gene1708<br>CTATACATGCAGTAGAACGCTGAAGGAAGGAGCTTGCTCTTTCCG<br>GATGAGTTGCGAACGGGTGAGTAACGCGTAGGTAACCTGCCTGGT<br>AGCGGGGGATAACTATTGGAAACGATAGCTAATACCGCATAACAG<br>TAGATATCGCATGATAGCTGCTTGAAAGGTGCAAATGCACCACTA<br>CCAGATGGACCTGCGTTGTATTAGCTAGTTGGTGAGGTAACGGCTC<br>ACCAAGGCAACGATACATAGCCGACCTGAGAGGGTGATCGGCCA<br>CACTGGGACTGAGACACGGCCCAGACTCCTACGGGAGGCAGCAGT<br>AGGGAATCTTCGGCAATGGACGGAAGTCTGACCGAGCAACGCCGC<br>GTGAGTGAAGAAGGTTTTCGGATCGTAAAGCTCTGTTGTAAGAGA<br>AGAACGAGTGTGAGAGTGGAAAGTTCACACTGTGACGGTATCTTA<br>CCAGAAAGGGACGGCTAACTACGTGCCAGCAGCCGCGGTAATAC<br>GTAGGTCCCGAGCGTTGTCCGGATTTATTGGGCGTAAAGCGAGCG<br>CAGGCGGTTAGATAAGTCTGAAGTTAAAGGCTGTGGCTTAACCAT<br>AGTACGCTTTGGAAACTGTTTAACTTGAGTGCAAGAGGGGAGAGT<br>GGAATTCCATGTGTAGCGGTGAAATGCGTAGATATATGGAGGAAC<br>ACCGGTGGCGAAAGCGGCTCTCTGGCTTGTAACTGACGCTGAGGC<br>TCGAAAGCGTGGGGAGCAAACAGGATTAGATACCCTGGTAGTCCA<br>CGCCGTAAACGATGAGTGCTAGGTGTTAGACCCTTTCCGGGGTTTA<br>GTGCCGTAGCTAACGCATTAAGCACTCCGCCTGGGGAGTACGACC<br>GCAAGGTTGAAACTCAAAGGAATTGACGGGGGCCCGCACAAGCG<br>GTGGAGCATGTGGTTTAATTCGAAGCAACGCGAAGAACCTTACCA<br>GGTCTTGACATCCCTCTGACCGCTCTAGAGATAGAGTTTTCCTTCG<br>GGACAGAGGTGACAGGTGGTGCATGGTTGTCGTCAGCTCGTGTCGT<br>GAGATGTTGGGTTAAGTCCCGCAACGAGCGCAACCCCTATTGTTAG<br>TTGCCATCATTGAGTTGGGCACTCTAGCGAGACTGCCGGTAATAAA<br>CCGGAGGAAGGTGGGGATGACGTCAAATCATCATGCCCCTTATGA<br>CCTGGGCTACACACGTGCTACAATGGTTGGTACAACGAGTCGCAA

	GCCGGTGACGGCAAGCTAATCTCTTAAAGCCAATCTCAGTTCGGAT<br>TGTAGGCTGCAACTCGCCTACATGAAGTCGGAATCGCTAGTAATC<br>GCGGATCAGCACGCCGCGGTGAATACGTTCCCGGGCCTTGTACAC<br>ACCGCCCGTCACACCACGAGAGTTTGTAACACCCGAAGTCGGTGA<br>GGTAACCTTTAGGAGCCAGCCGTCG
Streptococcus sp. 21WXBC0044M1	>21WXBC0044M1_gene1818<br>CTATACATGCAGTAGAACGCTGAAGGAGGAGCTTGCTTCTCTGGAT<br>GAGTTGCGAACGGGTGAGTAACGCGTAGGTAACCTGCCTGGTAGC<br>GGGGGATAACTATTGGAAACGATAGCTAATACCGCATAAGAGTAG<br>ATGTTGCATGACATTTGCTTAAAAGGTGCAATTGCATCACTACCAG<br>ATGGACCTGCGTTGTATTAGCTAGTTGGTGAGGTAACGGCTCACCA<br>AGGCGACGATACATAGCCGACCTGAGAGGGTGATCGGCCACACTG<br>GGACTGAGACACGGCCCAGACTCCTACGGGAGGCAGCAGTAGGG<br>AATCTTCGGCAATGGACGGAAGTCTGACCGAGCAACGCCGCGTGA<br>GTGAAGAAGGTTTTCGGATCGTAAAGCTCTGTTGTAAGAGAAGAA<br>CGAGTGTGAGAGTGGAAAGTTCACACTGTGACGGTATCTTACCAG<br>AAAGGGACGGCTAACTACGTGCCAGCAGCCGCGGTAATACGTAG<br>GTCCCGAGCGTTGTCCGGATTTATTGGGCGTAAAGCGAGCGCAGG<br>CGGTTAGATAAGTCTGAAGTTAAAGGCTGTGGCTTAACCATAGTAC<br>GCTTTGGAAACTGTTTAACTTGAGTGCAAGAGGGGAGAGTGGAAT<br>TCCATGTGTAGCGGTGAAATGCGTAGATATATGGAGGAACACCGG<br>TGGCGAAAGCGGCTCTCTGGCTTGTAACTGACGCTGAGGCTCGAA<br>AGCGTGGGGAGCAAACAGGATTAGATACCCTGGTAGTCCACGCCG<br>TAAACGATGAGTGCTAGGTGTTAGACCCTTTCCGGGGTTTAGTGCC<br>GCAGCTAACGCATTAAGCACTCCGCCTGGGGAGTACGACCGCAAG<br>GTTGAAACTCAAAGGAATTGACGGGGGCCCGCACAAGCGGTGGA<br>GCATGTGGTTTAATTCGAAGCAACGCGAAGAACCTTACCAGGTCTT<br>GACATCCCTCTGACCGCTCTAGAGATAGAGTTTTCCTTCGGGACAG<br>AGGTGACAGGTGGTGCATGGTTGTCGTCAGCTCGTGTCGTGAGATG<br>TTGGGTTAAGTCCCGCAACGAGCGCAACCCCTATTGTTAGTTGCCA<br>TCATTCAGTTGGGCACTCTAGCGAGACTGCCGGTAATAAACCGGA<br>GGAAGGTGGGGATGACGTCAAATCATCATGCCCCTTATGACCTGG<br>GCTACACACGTGCTACAATGGCTGGTACAACGAGTCGCAAGCCGG<br>TGACGGCAAGCTAATCTCTTAAAGCCAGTCTCAGTTCGGATTGTAG<br>GCTGCAACTCGCCTACATGAAGTCGGAATCGCTAGTAATCGCGGA<br>TCAGCACGCCGCGGTGAATACGTTCCCGGGCCTTGTACACACCGC<br>CCGTCACACCACGAGAGTTTGTAACACCCGAAGTCGGTGAGGTAA<br>CCTTTAGGAGCCAGCC
Streptococcus sp. BJSWXB5TM5	>BJSWXB5TM5_gene0977<br>GCGGCTGGCTCCTAAAAGGTTACCTCACCGACTTCGGGTGTTACAA<br>ACTCTCGTGGTGTGACGGGCGGTGTGTACAAGGCCCGGGAACGTA<br>TTCACCGCGGCGTGCTGATCCGCGATTACTAGCGATTCCGACTTCA<br>TGTAGGCGAGTTGCAGCCTACAATCCGAACTGAGACTGGCTTTAA<br>GAGATTAGCTTGCCGTCACCGACTTGCGACTCGTTGTACCAGCCAT<br>TGTAGCACGTGTGTAGCCCAGGTCATAAGGGGCATGATGATTTGA<br>CGTCATCCCCACCTTCCTCCGGTTTATTACCGGCAGTCTCGCTAGA<br>GTGCCCAACTAAATGATGGCAACTAACAATAGGGGTTGCGCTCGT<br>TGCGGGACTTAACCCAACATCTCACGACACGAGCTGACGACAACC<br>ATGCACCACCTGTCACCTCTGTCCCGAAGGAAAGCTCTATCTCTAG<br>AGCGGTCAGAGGGATGTCAAGACCTGGTAAGGTTCTTCGCGTTGCT<br>TCGAATTAAACCACATGCTCCACCGCTTGTGCGGGCCCCCGTCAAT

	TCCTTTGAGTTTCAACCTTGCGGTCGTACTCCCCAGGCGGAGTGCTT<br>AATGCGTTAGCTACGGCACTAAACCCCGGAAAGGGTCTAACACCT<br>AGCACTCATCGTTTACGGCGTGGACTACCAGGGTATCTAATCCTGT<br>TTGCTCCCCACGCTTTCGAGCCTCAGCGTCAGTTACAAGCCAGAGA<br>GCCGCTTTCGCCACCGGTGTTCCTCCATATATCTACGCATTTCACCG<br>CTACACATGGAATTCCACTCTCCCCTCTTGCACTCAAGTTAAACAG<br>TTTCCAAAGCGTACTATGGTTAAGCCACAGCCTTTAACTTCAGACT<br>TATCTAACCGCCTGCGCTCGCTTTACGCCCAATAAATCCGGACAAC<br>GCTCGGGACCTACGTATTACCGCGGCTGCTGGCACGTAGTTAGCCG<br>TCCCTTTCTGGTAAGATACCGTCACAGTGTGAACTTTCCACTCTCAC<br>ACTCGTTCTTCTCTTACAACAGAGCTTTACGATCCGAAAACCTTCTT<br>CACTCACGCGGCGTTGCTCGGTCAGACTTCCGTCCATTGCCGAAGA<br>TTCCCTACTGCTGCCTCCCGTAGGAGTCTGGGCCGTGTCTCAGTCCC<br>AGTGTGGCCGATCACCCTCTCAGGTCGGCTATGTATCGTCGCCTTG<br>GTGAGCCGTTACCTCACCAACTAGCTAATACAACGCAGGTCCATCT<br>GGTAGTGATGCAATTGCACCTTTCAAGCAGATATCATGCAATATCT<br>ACTGTTATGCGGTATTAGCTATCGTTTCCAATAGTTATCCCCCGCTA<br>CCAGGCAGGTTACCTACGCGTTACTCACCCGTTCGCAACTCATCCG<br>GAGAAGCAAGCTCCTCCTTCAGCGTTCTACTGCATGTATAG
Streptococcus sp. JSWX21MRM4	>JSWX21M4MR_gene1859<br>TTGCTTCTCCGGATGAGTTGCGAACGGGTGAGTAACGCGTAGGTAA<br>CCTGCCTGGTAGCGGGGGATAACTATTGGAAACGATAGCTAATAC<br>CGCATAACAGTAGATATTGCATGATATCTGCTTGAAAGGTGCAATT<br>GCATCACTACCAGATGGACCTGCGTTGTATTAGCTAGTTGGTGAGG<br>TAACGGCTCACCAAGGCAACGATACATAGCCGACCTGAGAGGGTG<br>ATCGGCCACACTGGGACTGAGACACGGCCCAGACTCCTACGGGAG<br>GCAGCAGTAGGGAATCTTCGGCAATGGACGGAAGTCTGACCGAGC<br>AACGCCGCGTGAGTGAAGAAGGTTTTCGGATCGTAAAGCTCTGTT<br>GTAAGAGAAGAACGAGTGTGAGAGTGGAAAGTTCACACTGTGAC<br>GGTATCTTACCAGAAAGGGACGGCTAACTACGTGCCAGCAGCCGC<br>GGTAATACGTAGGTCCCGAGCGTTGTCCGGATTTATTGGGCGTAAA<br>GCGAGCGCAGGCGGTTAGATAAGTCTGAAGTTAAAGGCTGTGGCT<br>TAACCATAGTACGCTTTGGAAACTGTTTAACTTGAGTGCAAGAGGG<br>GAGAGTGGAATTCCATGTGTAGCGGTGAAATGCGTAGATATATGG<br>AGGAACACCGGTGGCGAAAGCGGCTCTCTGGCTTGTAACTGACGC<br>TGAGGCTCGAAAGCGTGGGGAGCAAACAGGATTAGATACCCTGGT<br>AGTCCACGCCGTAACGATGAGTGCTAGGTGTTAGACCCTTTCCGGG<br>GTTTAGTGCCGTAGCTAACGCATTAAGCACTCCGCCTGGGGAGTAC<br>GACCGCAAGGTTGAAACTCAAAGGAATTGACGGGGGCCCGCACA<br>AGCGGTGGAGCATGTGGTTTAATTCGAAGCAACGCGAAGAACCTT<br>ACCAGGTCTTGACATCCCTCTGACCGCTCTAGAGATAGAGTTTTCC<br>TTCGGGACAGAGGTGACAGGTGGTGCATGGTTGTCGTCAGCTCGTG<br>TCGTGAGATGTTGGGTTAAGTCCCGCAACGAGCGCAACCCCTATTG<br>TTAGTTGCCATCATTTAGTTGGGCACTCTAGCGAGACTGCCGGTAA<br>TAAACCGGAGGAAGGTGGGGATGACGTCAAATCATCATGCCCCTT<br>ATGACCTGGGCTACACACGTGCTACAATGGCTGGTACAACGAGTC<br>GCAAGTCGGTGACGGCAAGCTAATCTCTTAAAGCCAGTCTCAGTTC<br>GGATTGTAGGCTGCAACTCGCCTACATGAAGTCGGAATCGCTAGT<br>AATCGCGGATCAGCACGCCGCGGTGAATACGTTCCCGGGCCTTGT<br>ACACACCGCCCGTCACACCACGAGAGTT
